# Improving family health climate, effect of role modeling and maternal support in female students

**DOI:** 10.1186/s12875-023-02015-7

**Published:** 2023-03-13

**Authors:** Jeyran Ostovarfar, Mohammad Hossein Kaveh, Hossein Molavi Vardanjani, Leila Ghahramani, Masoud Karimi, Abdolrahim Asadollahi, Razie Zare

**Affiliations:** 1 MPH Program, Medical School, Shiraz University of Medical Sciences, Setad Square, Shiraz, Fars Iran; 2 Department of Health Promotion, School of Health, Shiraz University of Medical Sciences, Shiraz, Iran; 3 Department of Industrial & Organizational Psychology, Faculty Education & Psychology, Shahid Chamran University, Ahvaz, Iran

**Keywords:** Mother, Family health climate, Nutrition, Physical activity

## Abstract

**Introduction:**

Girls can use their mother’s emotional, informational and behavioral support to perform healthy behaviors due to their constant access to their mothers. This study aimed to evaluate the effect of role modeling and maternal support in the family to improve healthy behaviors and perceived Family Health Climate (FHC) in female students.

**Methods:**

In this educational quasi-experimental study, 261 female students (133 in the intervention group and 128 in the control group) and 223 mothers (109 intervention and 114 control) were selected using the cluster multi-stages sampling method and entered the study. Participants (intervention and control groups) completed the FHC scale at three stages (before intervention, immediately after the intervention, and 2 months after intervention). A training program that comprised 12 sessions for students and six sessions for their mothers using collaborative learning techniques and printed materials was conducted with the experimental group. Also after completing the questionnaire in the follow-up phase, pamphlets and educational videos were given to the control group. Data were analyzed using SPSS20 via a chi-square test, independent t-test, and Repeated Measures ANOVA at a significance level of 0.05.

**Results:**

Before the intervention, there was no significant difference between demographic variables and the score of the FHC scale in both groups (*p* < 0.05). Immediately and 2 months after the intervention, the experimental group (female students and their mothers) showed a significant increase in dimensions of FHC, including FHC-NU (Family Health Climate-Nutrition) and FHC-PA (Family Health Climate-Physical Activity), compared to the control group (*p* < 0.05).

**Conclusions:**

Educating and informing mothers about the impact of their role modeling on their children, especially girls, can make them more aware of health-oriented behaviors towards their children. Such findings reinforced the importance of focusing on actions to encourage a healthy lifestyle (healthy diet and physical activity) in students with a focus on role modeling and parental support, especially mothers.

**Supplementary Information:**

The online version contains supplementary material available at 10.1186/s12875-023-02015-7.

## Background

Well-being and positive social functioning are rooted in childhood. Perhaps the strongest predictor of these paths is the family quality of life and the parental environment in which the child is placed [1, 2]. Adolescents’ health behaviors are influenced by social contexts, such as peer groups, the school environment, and the family. Because of its continual effect on children and adolescents’ physical activity and health behaviors, the family is critical [3].

The family is the core of the socialization of members, a place where values are passed on to individuals, ideas are learned and adopted, and beliefs and norms of behavior are acquired. Its members are divided into separate subsystems through symbolic boundaries; each contributes to the family’s functioning by performing the necessary roles and responsibilities [3, 4]. According to the family system’s approach, the family is more than the sum of individuals. This theoretical framework requires interactions within and between individuals and the shared family environment. Therefore, the family environment’s characteristics should affect individual behavior and factors. One aspect of the family environment may be the family health climate [3, 5].

Family health climate (FHC): Family health climate is defined as shared perceptions and cognitions about a healthy lifestyle within the family. It reflects the individual experience of daily family life, evaluating health-related issues and expectations according to common values, routine behaviors, and family interaction patterns. The FHC acts as a framework for health-related behaviors, is the basis for regulating health-related behaviors, and provides a reference for evaluating and interpreting individual behaviors. Thus, the FHC is an aspect of the family environment that shapes the daily health behaviors of family members. FHC can be assessed for healthy eating using the FHC-Nutrition scale and physical activity using the FHC-Physical Activity scale. FHC represents a variable at the family level related to internal and interpersonal relationships within the family environment and individual factors [5].

Family health climate has been demonstrated to affect the weekly physical activity and the consumption of nutritious foods in adolescents and children [3, 6]. A *positive family health climate* is an environment where eating healthfully and being physically active are highly valued and an integral part of daily family life [5]. While the strong association between physical activity and nutrition with health has been proven [7, 8]. She family health climate model can be utilized to understand how an individual’s family can shape their health behaviors [5].

Parents and children are part of a family whose members influence each other’s behavior and this influence is mutual [9]. It has been reported that due to the ongoing communication between parents and children’s behaviors and attitudes, parents’ modeling impacts how children think and behave about food and physical activity [10]. Parents also play a crucial role in transmitting health information and supporting their children’s healthy behavior [11]. Meanwhile, Wunsch et al.’s study shows that targeting all family members facilitates behavior change at the individual and family level, because the implemented strategies address changes in daily family life [12].

Mothers are primary caregivers who usually provide a framework for children’s meals with specific foods and show them how much they can eat [13–15]. On the other hand, numerous studies show that a mother’s role modeling healthy active behaviors has a more impact on children than the paternal model [16, 17]. Girl’s health behaviors align with their mothers for several reasons; girls eat the food that their mothers prepare for them [18], they at an early age are more likely to pattern from their parents’ eating behaviors [19], and children with active mothers are more likely to be active than children with non-active mothers [20]. Children’s preferences for certain foods usually reflect eating at home [19]. Accordingly, researchers consider mothers to be the leading cause of change in influencing their children’s diet and physical activity [21] and mothers who are trained in healthy eating and physical activity are more likely to engage in health-oriented behaviors for their children [19]. Also, compared to other parent-child relationships, interdependence and emotional dependence in the mother-daughter relationship is more substantial, and the nature of the relationship plays a vital role in girls’ social and psychological well-being. Therefore, girls can use their emotional, informational and behavioral support to perform healthy behaviors due to their constant access to their mothers [22].

This study aimed to evaluate the effect of maternal support as role modeling in the family to improve healthy behaviors in girls. Furthermore, was done in answer to this question, is the mother’s support intervention as role modeling effective in changing the family health climate perceived by female students and their mothers?

## Methods

### Research design

The purpose of this study was to evaluate the impact of the educational program on students and their mothers, using role modeling and mothers’ support to promote a perceived family health climate in female students and their mothers. Based on this goal, a quasi-experimental plan consisting of an intervention group and a control group was approved in which pre-test, post-test, and 2-month follow-up were designed to evaluate the program’s effects. Ethics approval was obtained from research ethics board of the Shiraz University of Medical Sciences. The study was approved by the ethics committee on 07/08/2019 (IR.SUMS.REC.1398.896).

### Participants

Participants in this study were fifth-grade female students who studied in Shiraz schools and their mothers. Sampling was done by the cluster sampling method among the four education districts of Shiraz, each of which has 43, 39, 58, and 55 schools, respectively. From the four districts, two districts were randomly selected (districts 2 and 4). Schools in each area (four schools) were randomly allocated to experimental and control groups. Two classes were randomly selected in each school, and 261 female students were eventually selected.

In the data collection process, the first step was to visit the selected schools, meet with school principals and inform them about the content and purpose of the study. After receiving the approval of the school principals, the pre-test questionnaire form was completed by 261 fifth-grade students who met the inclusion criteria. In contrast, the questionnaire forms for mothers in the distribution envelopes were sent to them by the children. After three working days, the researchers returned to the schools, and the questionnaire forms completed by the mothers were collected. Mothers who participated in the intervention program meant that they completed the questionnaire themselves. Based on the collected data, mothers’ questionnaires were considered for further analysis. Two hundred twenty-three mothers finally agreed to participate in the study by filling out questionnaires after calling and explaining the objectives and content of the research. Among the mothers who decided to participate in the program, (109 intervention) and (114 control) were divided into two groups based on the division of their children’s schools and classes. At the beginning of the intervention, the educational and occupational levels of the mothers were checked in terms of the homogeneity of the intervention and control groups, because it was thought that these factors affect the results of the intervention.

The demographic characteristics of the students in the intervention and control groups are presented in Table 1.

**Table 1:** Demographic characteristics of students in the two groups of intervention and control

Demographic variables		Number of people in the intervention group	Number of people in the control group	***p***-value *
**Mother’s education**	Primary	5	3	0.113
	High school	9	3	
	Diploma	33	36	
	Bachelor	66	72	
	Master degree and higher	20	14	
**Father’s education**	Primary	8	3	0.235
	High school	7	7	
	Diploma	39	48	
	Bachelor	54	56	
	Master degree and higher	25	14	
**Mother’s occupation**	Employee	50	41	0.158
	housewife	72	82	
	self-employment	11	5	
	Retired	0	0	
**Father’s occupation**	Employee	64	52	0.480
	worker	5	9	
	self-employment	57	60	
	Retired and unemployed	7	7	
**Number of family members**	3	33	24	0.639
	4	70	72	
	5–6	29	30	
	7 and more	1	2	

The inclusion criteria for female students and their mothers were: to be studying in public schools in Shiraz, their grade of education should be fifth, to complete the written informed consent form by students and their mothers, and the exclusion criteria were: absenteeism in educational sessions for two sessions or more, refusing to continue participating in the project, leave the research environment (such as changing schools, etc.).

### Instruments

Data collection tools included a demographic questionnaire and a family health climate scale.

The demographic questionnaire included the student’s age, parental occupation, parental education, and the number of family members.

The Family Health Climate scale was developed by Niermann et al. in 2014 to assess family members’ health behaviors. The questions of the FHC includes two separate scales: FHC-NU consists of 17 questions with four subscales (value, for example, “a healthy diet plays an important role in our lives”; communication, for example, “we talk about which foods are healthy”; cohesion, for example, “we appreciate spending time together during meals”; and consensus, for example, “we rarely argue about food- or diet-related matters”). All questions were begun with “In our family … ,” [5].

FHC-PA contains 14 questions with three subscales (value, for example, “it is normal in our family to bephysically active in our leisure time”; cohesion, for example, “… we have fun doing physical activitie stogether (e.g.,bike tours and hikes)”; and information, for example, “we collect information (e.g., on the internet) on physical activity and exercise”) [5].

Answers were given on a four-point rating scale (0 = “definitely false,” 1 = “rather false,” 2 = “rather true,” and 3 = “definitely true”). In a study by Niermann et al., mothers, fathers, and adolescents completed a questionnaire separately. The internal correlation was *α*FHC-PA = 0*:*92 and *α*FHC-NU = 0*:*86 for the mothers, *α*FHC-PA = 0*:*90 and *α*FHC-NU = 0*:*86 for the fathers, and *α*FHC-PA = 0*:*90 and *α*FHC-NU = 0*:*85 for the adolescents [7].

In the Persian version of the FHC-Scale, Cronbach’s alpha coefficient for FHC-PA in female students and their mothers was 0.88 and 0.86 for the whole scale. Cronbach’s alpha coefficient for FHC-NU in female students and their mothers was 0.83 and 0.92 for the whole scale [23].

The education program for students and their mothers was implemented with the permission of the school administration. The program included 12 sessions for students and six sessions for their mothers.

### Procedure and program

In the student sessions, there were six sessions related to healthy eating education and promoting eating behaviors. At the beginning of the sessions, students received healthy snacks such as fruit, milk, or healthy pre-prepared foods. During the sessions, various methods were used, such as; lectures on general topics, use of audio-visual presentation, questions and answers, problem-solving, sample cases, asking students to make a list of healthy meals with the help of mothers, as well as making list of meals that students usually prefer to use and then a discussion was held about it. Six sessions related to physical activity were performed by doing the students’ favorite sports with the cooperation and presence of a physical activity instructor. Also, during the sessions about the minimum physical activity required by adolescents and the definition of moderate to vigorous physical activities and types of sports such as endurance, stretching, etc. were educated through educational videos, lectures, and booklets. The research team also coordinated with a sports club to facilitate the intervention group’s enrollment in a sports class.

In the mothers’ group, due to the busy schedule of mothers, the number of sessions was less, so more nutritional information and physical activity were provided to mothers in the form of short texts through social media. Three nutrition education sessions were conducted to get acquainted with the nutrition groups as well as the nutritional needs of adolescents through lectures, questions, answers, audio-visual presentations, and brainstorming. Mothers were also encouraged to cooperate and advise on preparing their children’s meal lists and involving children in choosing food when shopping. Three physical activity sessions were held. In the first session, the researchers gave a speech to express the objectives of the physical activity sessions, obtain the mothers’ consent to participate in the classes, and attend a sports club that was previously coordinated. Also, they were encouraged to start sports that did not require special equipment to have regular physical activity. Also, physical activity training videos that were appropriate for mothers and their daughters were shown. They were asked to organize family walks in their free time, encourage children and other family members to exercise, act as role models, and provide their children with the necessary facilities for physical activity.

To reduce dropout rates in the intervention stage, a reminder SMS was sent to the mothers of the intervention group before the training sessions. Invitation letters were also sent to mothers through students. Because the students were in school, one of the researchers encouraged them to attend the training sessions.

In the control group, the questionnaires were completed in 3 stages. When completing the questionnaires, healthy meals were given to students and their mothers, and after completing the questionnaire, educational booklets and videos were given to them in the follow-up stage.

### Statistical analysis

The collected data were analyzed using SPSS version 20. A Chi-square test was used to check the homogeneity of demographic variables in the control and intervention groups and a t-test was used to check the mean of the student’s age in the two groups. After that, an independent t-test and Repeated Measurement Anova at the significance level of 0.05 to examine the impact of the intervention on FHC -NU and its variables (Communication, Value, Cohesion, Consensus), FHC -PA and its variables (Information, Cohesion, Value) in students and their mothers were done.

### Findings

A total of 261 students were included in the study, including 133 in the intervention group and 128 in the control group. As shown in Table 1, the Chi-square test did not show a significant difference between the intervention and control groups regarding demographic variables (*p* < 0.05). In other words, the two groups were identical in these characteristics. The results of the t-test showed no statistically significant difference between the mean of the student’s age in the intervention and control groups (*p* = 0.254). The means of students’ age in the intervention and control groups were 19.57 and 10.50 years, respectively.

The results showed that the dimensions of FHC, including FHC-NU and FHC-PA (their subscales) in the sample of students in the intervention group after the intervention, were significantly different from before the educational intervention. However, in the control group, this difference was not significant. At the beginning of the intervention, there was no significant difference in the mean scores of FHC dimensions and their subscales between the two groups, but immediately after the intervention and 2 months after the intervention (follow-up stage), there was a significant difference (Table 2).

**Table 2:** Comparison of the mean score of FHC in the two groups of intervention and control before the intervention, immediately after the intervention, and 2 months after the educational intervention in female students

Variable	Group	Before intervention M ± SD	Immediately after the intervention	Two month after intervention M ± SD	***P***-Value^**٭٭**^
**FHC-NU**	experimental	27.85 ± 10.38	39.14 ± 9.47	32.58 ± 11.71	> 0.001
	control	28.40 ± 9.45	29.50 ± 10.19	28.27 ± 11.23	0.614
	*P*-Value٭	0.876	> 0.001	0.005	
**Communication**	experimental	7.37 ± 4.38	10.50 ± 4.31	8.73 ± 4.19	> 0.001
	control	7.34 ± 4.33	7.75 ± 4.23	7.50 ± 4.10	0.759
	*P*-Value٭	0.774	> 0.001	0.027	
**Value**	experimental	6.61 ± 3.23	10.56 ± 2.36	8.58 ± 2.96	> 0.001
	control	6.67 ± 3.60	7.43 ± 3.59	6.96 ± 3.60	0.190
	*P*-Value٭	0.841	> 0.001	> 0.001	
**Cohesion**	experimental	10.57 ± 4.02	12.99 ± 3.43	10.69 ± 4.24	> 0.001
	control	10.86 ± 3.71	10.90 ± 4.39	9.86 ± 4.14	0.122
	*P*-Value٭	0.881	> 0.001	0.133	
**Consensus**	experimental	3.30 ± 2.81	5.18 ± 2.89	4.58 ± 2.46	> 0.001
	control	3.47 ± 2.97	3.49 ± 2.70	3.95 ± 2.30	0.151
	*P*-Value٭	0.734	> 0.001	0.046	
**FHC-PA**	experimental	18.09 ± 11.14	27.37 ± 11.02	22.00 ± 9.65	> 0.001
	control	18.98 ± 11.66	20.43 ± 11.12	19.52 ± 9.58	0.802
	*P*-Value٭	0.527	> 0.001	0.052	
**Information**	experimental	4.08 ± 4.18	6.88 ± 4.35	5.43 ± 3.54	> 0.001
	control	4.62 ± 4.29	5.27 ± 3.92	4.83 ± 3.08	0.378
	*P*-Value٭	0.477	0.008	0.176	
**Cohesion**	experimental	7.38 ± 5.24	11.79 ± 3.59	8.98 ± 4.44	> 0.001
	control	8.00 ± 4.79	8.36 ± 4.45	7.97 ± 4.52	0.736
	*P*-Value٭	0.227	> 0.001	0.053	
**Value**	experimental	7.21 ± 5.10	11.35 ± 3.75	9.57 ± 4.21	> 0.001
	control	7.07 ± 4.92	8.62 ± 4.49	8.31 ± 4.38	0.158
	*P*-Value٭	0.812	> 0.001	0.028	

٭Independent t-test ٭٭ Repeated Measurement.

## Result of intervention in mothers

The results of the t-test showed no statistically significant difference between the mean of the mother’s age in the intervention and control groups (*p* = 0.061). The means of mother’s age in the intervention and control groups was 38.94 and 37.47 years, respectively. Similarly, the mean age of students’ fathers in the intervention group was 42.95, and in the control group was 42.40 years. The independent t-test showed that the two groups were homogeneous(*p* = 0.457). There was no significant difference between the two groups of intervention and control before the educational intervention, as shown in Table 1 in terms of the level of education of the mother and father, parent’s occupation status, and the number of family members, so the two groups were homogeneous in terms of these variables.

The results showed that the dimensions of FHC, including FHC-NU and FHC-PA (their subscales) in the sample of mothers in the intervention group, differed significantly from before the educational intervention. However, in the control group, this difference was not significant. At the beginning of the intervention, there was no significant difference in the mean scores of FHC dimensions and their subscales between the two groups of mothers, but immediately after the intervention and in the follow-up stage, there was a significant difference (Table 3).

**Table 3:** Comparison of the mean score of FHC in the two groups of intervention and control before the intervention, immediately after the intervention and 2 months after the educational intervention in mothers

Variable	Group	Before intervention M ± SD	Immediately after the intervention	Two month after intervention M ± SD	***P***-Value^**٭٭**^
**FHC-NU**	experimental	25.72 ± 8.43	41.02 ± 7.27	34.69 ± 11.71	> 0.001
	control	25.78 ± 9.04	28.91 ± 12.32	26.44 ± 12.84	0.120
	*P*-Value٭	0.961	> 0.001	> 0.001	
**Communication**	experimental	6.64 ± 3.70	12.34 ± 2.56	9.92 ± 3.85	> 0.001
	control	6.88 ± 3.74	7.92 ± 4.25	7.13 ± 3.98	0.126
	*P*-Value٭	0.817	> 0.001	> 0.001	
**Value**	experimental	6.62 ± 2.38	9.82 ± 2.33	8.54 ± 2.69	> 0.001
	control	6.79 ± 2.61	7.51 ± 3.60	6.80 ± 3.54	0.142
	*P*-Value٭	0.628	> 0.001	> 0.001	
**Cohesion**	experimental	8.63 ± 3.55	12.84 ± 2.67	11.01 ± 3.50	> 0.001
	control	8.46 ± 3.77	9.29 ± 4.40	8.22 ± 4.73	0.213
	*P*-Value٭	0.724	> 0.001	> 0.001	
**Consensus**	experimental	3.83 ± 2.52	6.02 ± 2.25	5.22 ± 2.33	> 0.001
	control	3.65 ± 2.43	4.19 ± 2.43	4.29 ± 2.50	0.153
	*P*-Value٭	0.884	> 0.001	0.029	
**FHC-PA**	experimental	19.51 ± 8.96	27.01 ± 6.66	22.47 ± 8.50	> 0.001
	control	19.62 ± 8.86	22.36 ± 8.69	21.36 ± 8.95	0. 229
	*P*-Value٭	0.924	> 0.001	0.344	
**Information**	experimental	4.31 ± 3.39	6.72 ± 2.82	4.68 ± 3.07	> 0.001
	control	4.86 ± 3.45	5.56 ± 3.34	5.58 ± 3.09	0.297
	*P*-Value٭	0.812	0.009	0.052	
**Cohesion**	experimental	7.67 ± 3.91	10.17 ± 4.45	8.22 ± 4.44	> 0.001
	control	7.47 ± 3.85	8.20 ± 4.75	7.47 ± 4.52	0.575
	*P*-Value٭	0.727	> 0.001	0.197	
**Value**	experimental	7.53 ± 4.12	10.11 ± 2.87	9.57 ± 4.21	> 0.001
	control	7.29 ± 4.05	8.60 ± 3.82	8.31 ± 4.38	0.060
	*P*-Value٭	0.689	0.002	0016	

٭Independent t-test

٭٭ Repeated Measurement

## Discussion

We investigated the effect of role modeling program training and mothers’ support on the perceived family health climate level in female students and their mothers compared to the control group (without support program and role modeling). (See attached Supplementary Fig. 1).

Because of the researcher’s relationship with the students, all students participated in the study, and 196 (75%) mothers participated based on the inclusion criteria and interests.

Considering that the maximum possible score of FHC-NU is 51, the study found that the average scores before intervention for the children (approximately 27) and mothers (approximately 25) in the intervention and control groups were low. The low pre-intervention scores are thought to be since the study sample was selected from a general population that had not experienced nutrition and physical activity interventions.

In the current study, as the results section shows, the changes during the study in the dimensions of FHC-NU (Communication, Value, Cohesion, Consensus) and FHC-PA (Information, Cohesion, Value) in the group of students are in line with the changes in the group of mothers. It seems that this information confirms the study results of Herman et al. [20].

The process of the present study is similar to a study that showed that family participation, especially mothers, in a school-based intervention is an important component of the program [18]. However, this study contributes to a growing body of literature emphasizing the importance of family and maternal involvement in influencing their children’s nutritional status and physical activity [19–21]. This may be because mothers act as important role models and comprehensive advocates of their child’s eating and physical activity behaviors [24], so it is reasonable to ask them to participate in programs on these critical issues actively. Also, comparing the results of the data obtained from the amount of students physical activity and their mothers during the present study shows that with the increase in the amount of mothers physical activity in the intervention phase as a role model, the amount of students physical activity has also increased and with reduction in the amount of mothers physical activity in the follow-up stage, the students physical activity has also decreased.

According to Goodwin et al.’s research, in many communities, women have become socialized as caregivers and maintainers of the family unit by preparing food, caring for family members, and talking to children to become productive adults [25]. Therefore, it seems that the results of the present study also confirm this assertion, and women can be considered promoters of family health, especially in developing countries where mothers’ lives are very closely related to their children’s lives [25]. Therefore, by improving the perceived family health climate in mothers, both in the field of nutrition and physical activity, positive results can be seen in the perceived health climate of other family members, especially girls who have a closer relationship with their mothers.

This is while Elizabeth et al. [26], in their study, argue that mothers try to achieve health goals (physical activity and healthy diet) for their family members, especially children, even if those goals are incompatible with the mothers’ living conditions. Of course, they also point out that when these cares and goals are considered as the usual duties of women, and all duties (the duties of mothers about improving the nutrition of family members and sports habits, buying food, cooking, taking children to sports training) is on the responsibility of a family member, i.e., mother, it will be harmful. Therefore, it seems that the long-term results of such interventions on mothers should be investigated, and more accurate results should be reported.

The follow-up phase of the study was associated with the Covid-19 prevalence epidemic, so it seems the decrease in physical activity in both groups can be related to the prevalence of the Covid-19 epidemic. As Shahidi et al., In their study, points out that the Covid-19 prevalence epidemic has limited the amount of physical activity in this period [27]. Robinson et al. Also reported negative changes in eating and physical activity during the prevalence of the Covid-19 epidemic among UK adults, which is consistent with the results of the present study [28].

However, in addition to paying attention to external factors (positive interferer factors such as training and encouragement to have a healthy diet and physical activity or negative interfering factors such as the prevalence of Covid-19), it seems better to pay attention to the psychosocial factors of individuals and interactions within their family [29]. In this regard, Naisseh et al. [30] also showed that the level of self-determination of parents about participation in physical activity is related to their support of their children’s physical activity, which can act as a positive or negative role model.

## Conclusion

The results of the present study showed that despite the decrease in the amount of mothers FHC-PA and its subscales in the follow-up and epidemic stage of COVID-19, it did not reduce the amount of female students FHC-PA and its subscales, which can be referred to as the supportive role of mothers. They have supported their children’s health-oriented behaviors by enduring the stress of the COVID-19 outbreak. Such findings reinforced the importance of focusing on actions to encourage a healthy lifestyle (healthy diet and physical activity) in students with a focus on parental role modeling and support, especially mothers. Also, educating and informing mothers about the impact of their role modeling on their children, especially girls, can make them more aware of health-oriented behaviors towards their children.

## Supplementary Information

**Additional file 1: Supplementary figure 1.** Changes in mean scores of FHC in the two groups of intervention and control in students and their mothers during the study.

## Data Availability

The datasets used and/or analyzed during the current study are available from the corresponding author upon reasonable request.

## Abbreviation

FHC: Family health climate
