# Supplementary material for: Improving family health climate, effect of role modeling and maternal support in female students
Source: BMC Prim Care. 2023 Mar 13;24:70. doi: 10.1186/s12875-023-02015-7 (PMC10008707; doi:10.1186/s12875-023-02015-7)
Supplement: Supplementary file 1 — Additional file 1: Supplementary figure 1. Changes in mean scores of FHC in the two groups of intervention and control in students and their mothers during the study. [file 12875_2023_2015_MOESM1_ESM.docx]

**Improving family health climate, effect of role modeling and maternal support in female students**


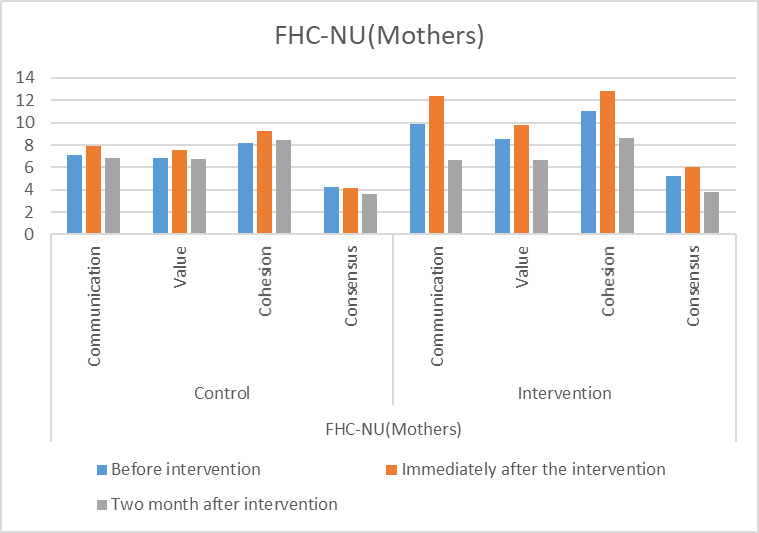

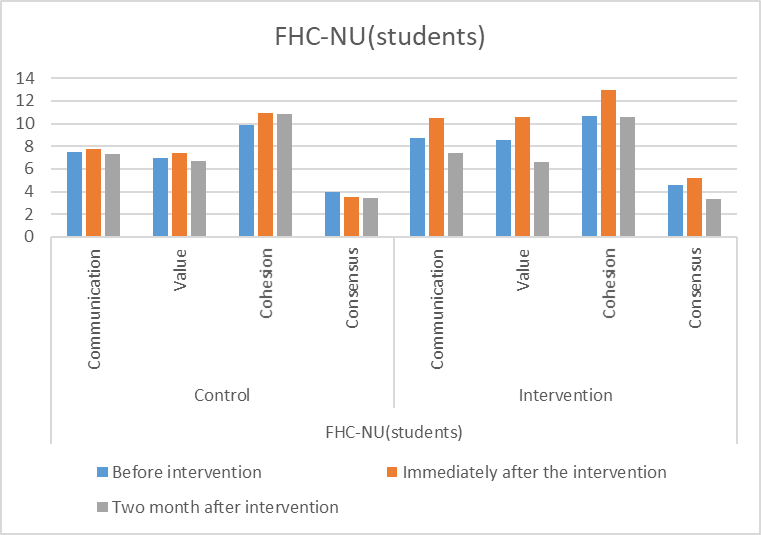

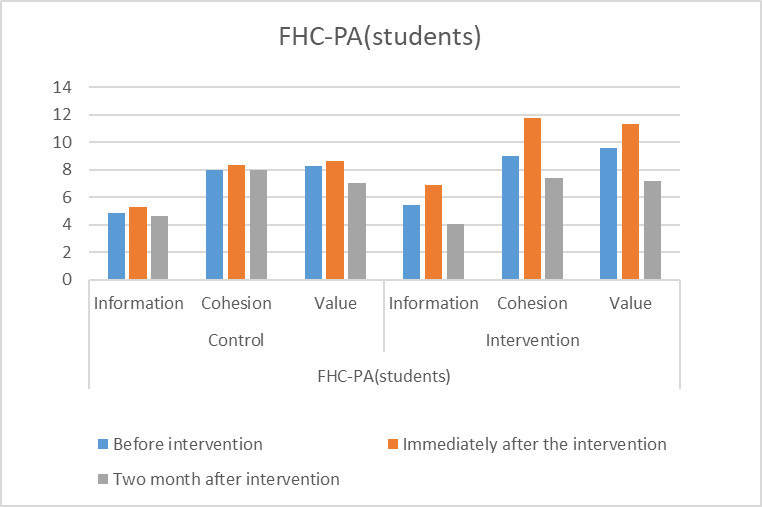

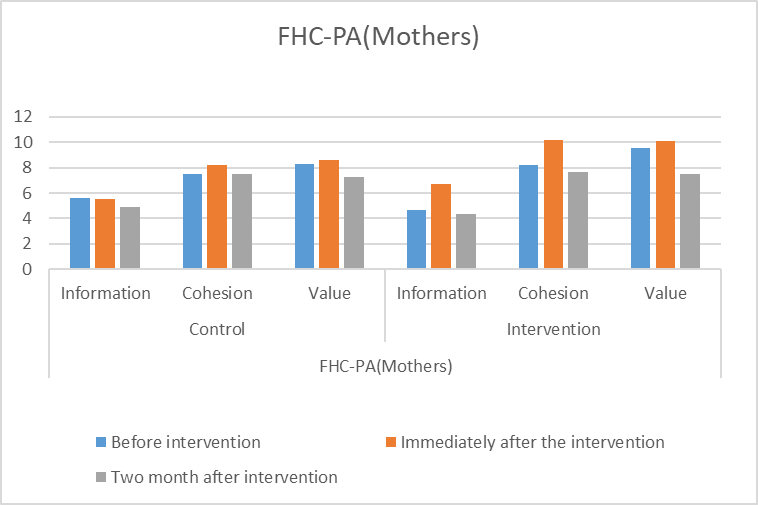


**Supplementary figure**.1 Changes in mean scores of FHC in the two groups of intervention and control in students and their mothers during the study
